# Phenyl 3,5-di-*tert*-butyl-2-hy­droxy­benzoate

**DOI:** 10.1107/S1600536810044028

**Published:** 2010-11-24

**Authors:** Alexander Carreño, Marcelo Preite, Juan Manuel Manriquez, Andrés Vega, Ivonne Chavez

**Affiliations:** a Programa de Doctorado, Fisico-Química Molecular, Universidad Andres Bello, Avenida República 275 Segundo Piso, Santiago, Chile; b Departamento de Química Orgánica, Facultad de Química, Pontificia Universidad Católica, Santiago, Chile; c Casilla 306 Correo 22, Santiago, Chile; d Departamento de Ciencias Químicas, Facultad de Ecología y Recursos Naturales, Universidad Andres Bello, Avenida República 275 3^er^ Piso, Santiago, Chile; e Centro para el Desarrollo de la Nanociencia y la Nanotecnología, CEDENNA, Chile

## Abstract

The title mol­ecule, C_21_H_26_O_3_, has a six-membered planar carbon ring as the central core, substituted at position 1 with phen­oxy­carbonyl, at position 2 with hy­droxy and at positions 3 and 5 with *tert*-butyl groups. The structure shows two independent but very similar mol­ecules within the asymmetric unit. For both independent mol­ecules, the ester carboxyl­ate group is coplanar with the central core, as reflected by the small C—C—O—C torsion angles [179.95 (17) and 173.70 (17)°]. In contrast, the phenyl substituent is almost perpendicular to the carboxyl­ate –CO_2_ fragment, as reflected by C—O—C—C torsion angles, ranging from 74 to 80°. The coplanarity between the central aromatic ring and the ester carboxyl­ate –CO_2_– group allows the formation of an intra­molecular hydrogen bond, with O⋯O distances of 2.563 (2) and 2.604 (2) Å.

## Related literature

For the synthesis of the title compound, see: Moore *et al.* (2008 ▶); Benisvy *et al.* (2004 ▶). For similar mol­ecules, see: Baptista (1966 ▶); Bilgram *et al.* (1982 ▶); Hammond *et al.* (2002 ▶).
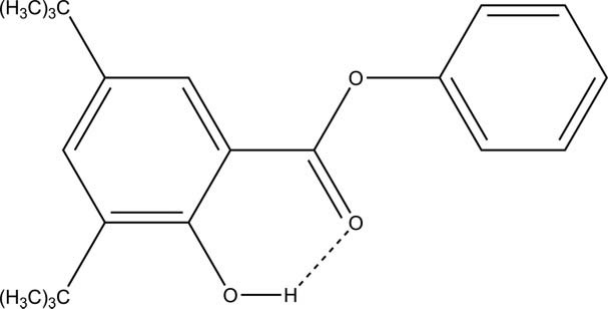


## Experimental

### 

#### Crystal data

C_21_H_26_O_3_
*M* *_r_* = 326.42
Triclinic, 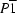

*a* = 10.5691 (11) Å
*b* = 12.2590 (13) Å
*c* = 15.0534 (16) Å
α = 96.400 (2)°
β = 93.813 (2)°
γ = 92.728 (2)°
*V* = 1931.0 (4) Å^3^
*Z* = 4
Mo *K*α radiation
μ = 0.07 mm^−1^
*T* = 297 K
0.50 × 0.21 × 0.20 mm

#### Data collection

Siemens SMART CCD area-detector diffractometer
Absorption correction: multi-scan (*SADABS*; Bruker, 2001 ▶) *T*_min_ = 0.964, *T*_max_ = 0.985
12058 measured reflections
6736 independent reflections
4140 reflections with *I* > 2σ(*I*)
*R*_int_ = 0.023

#### Refinement

*R*[*F* ^2^ > 2σ(*F* ^2^)] = 0.053
*wR*(*F* ^2^) = 0.153
*S* = 1.01
6736 reflections
445 parameters
H-atom parameters constrained
Δρ_max_ = 0.17 e Å^−3^
Δρ_min_ = −0.12 e Å^−3^

### 

Data collection: *SMART-NT* (Bruker, 2001 ▶); cell refinement: *SAINT-NT* (Bruker, 1999 ▶); data reduction: *SAINT-NT*; program(s) used to solve structure: *SHELXTL-NT* (Sheldrick, 2008 ▶); program(s) used to refine structure: *SHELXTL-NT*; molecular graphics: *SHELXTL-NT*; software used to prepare material for publication: *SHELXTL-NT*.

## Supplementary Material

Crystal structure: contains datablocks I, global. DOI: 10.1107/S1600536810044028/om2371sup1.cif

Structure factors: contains datablocks I. DOI: 10.1107/S1600536810044028/om2371Isup2.hkl

Additional supplementary materials:  crystallographic information; 3D view; checkCIF report

## Figures and Tables

**Table 1:** Hydrogen-bond geometry (Å, °)

*D*—H⋯*A*	*D*—H	H⋯*A*	*D*⋯*A*	*D*—H⋯*A*
O1—H1⋯O2	0.82	1.83	2.563 (2)	148
O4—H4⋯O5	0.82	1.88	2.604 (2)	147

**Fig. 1.**
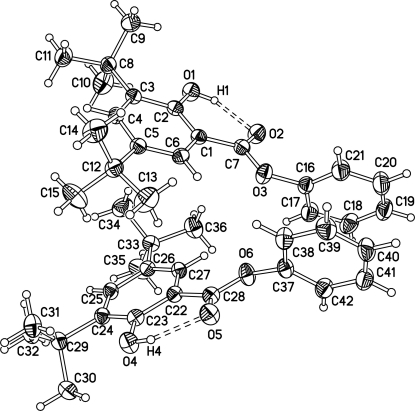
Molecular structure diagram showing the two independent molecules of I within the crystal, with atom numbering scheme. Displacement ellipsoids are at 25% probability level and H atoms are shown as spheres of arbitrary radii. Intramolecular hydrogen bonds are shown using a dotted line.

## supplementary crystallographic information

### Comment

Benzoate phenyl esters have been described to be precursors of benzimidazole molecules by reaction with 1,2-phenylenediamine (Moore *et al.*, 2008). The crystal shows two independent but very similar molecules within the asymmetric unit. For both independent molecules, the carboxylic acid group from ester is coplanar with the central core, as reflected by the small C—C—O—C torsion angles (C1—C7—O3—C16, 179.95 (17)°; C22—C28—O6—C37 173.70 (17)°), while the phenyl substituent is almost perpendicular to the carboxylate CO_2_ fragment (C7—O3—C16—C21 110.4 (2)°; C7—O3—C16—C17 - 74.1 (3)° and C28—O6—C37—C38 - 69.2 (3)°; C28—O6—C37—C42 117.4 (2)°). The co-planarity between the central aromatic ring and the carboxylate CO_2_ group from ester allows the definition of a intramolecular hydrogen bond, with O···O 2.563 (2) and 2.604 (2) Å.

The structure is closely related to that of the unsubstituted 2-hydroxybenzoic acid phenyl ester (Baptista, 1966; Bilgram *et al.* 1982; Hammond *et al.*, 2002), where the carboxylate group is almost coplanar to the phenyl ring where is attached (C—C—O—C less than 2° deviated from 180°) and the benzoate phenyl almost perpendicular to the carboxylate (C—O—C—C 75.8° and -100.5°).

The phenyl rings from the benzoate from each of the two molecules within the asymmetric unit defines a weak π···π interaction with *Cg*^1^(C16, C17, C18, C19, C20, C21)···*Cg*^2^(C37, C38, C39, C40, C41, C41) 3.903 (2) Å].

### Experimental

The compound was prepared by methods described in literature (Benisvy *et al.*, 2004) slighty modified by using CHCl_3_ for crystallization instead of pentane. The title compound was prepared in a 40% yield.

### Refinement

The H-atoms positions were calculated after each cycle of refinement using a riding model for each structure, with C—H distances in the range 0.93 to 0.96 Å. *U*_iso_(H) values were set equal to 1.5*U*_eq_ of the parent carbon atom for methyl groups and 1.2*U*_eq_ for the others. The hydroxyl hydrogen atoms were located in the difference Fourier map, but were subsequentely refined with constraints, O—H 0.82 Å and *U*_iso_(H) 1.5*U*_eq_ of the parent oxygen atom.

### Crystal data

**Table:**

C_21_H_26_O_3_	*Z* = 4
*M**_r_* = 326.42	*F*(000) = 704
Triclinic, *P*1	*D*_x_ = 1.123 Mg m^−^^3^
Hall symbol: -P 1	Mo *K*α radiation, λ = 0.71073 Å
*a* = 10.5691 (11) Å	Cell parameters from 2560 reflections
*b* = 12.2590 (13) Å	θ = 2.3–22.7°
*c* = 15.0534 (16) Å	µ = 0.07 mm^−^^1^
α = 96.400 (2)°	*T* = 297 K
β = 93.813 (2)°	Colourless, block
γ = 92.728 (2)°	0.50 × 0.21 × 0.20 mm
*V* = 1931.0 (4) Å^3^	

### Data collection

**Table:**

Siemens SMART CCD area-detector diffractometer	6736 independent reflections
Radiation source: fine-focus sealed tube	4140 reflections with *I* > 2σ(*I*)
graphite	*R*_int_ = 0.023
φ and ω scans	θ_max_ = 25.0°, θ_min_ = 2.3°
Absorption correction: multi-scan (*SADABS*; Bruker, 2001)	*h* = −12→12
*T*_min_ = 0.964, *T*_max_ = 0.985	*k* = −14→14
12058 measured reflections	*l* = −17→17

### Refinement

**Table:**

Refinement on *F*^2^	Primary atom site location: structure-invariant direct methods
Least-squares matrix: full	Secondary atom site location: difference Fourier map
*R*[*F*^2^ > 2σ(*F*^2^)] = 0.053	Hydrogen site location: inferred from neighbouring sites
*wR*(*F*^2^) = 0.153	H-atom parameters constrained
*S* = 1.00	*w* = 1/[σ^2^(*F*_o_^2^) + (0.0783*P*)^2^] where *P* = (*F*_o_^2^ + 2*F*_c_^2^)/3
6736 reflections	(Δ/σ)_max_ < 0.001
445 parameters	Δρ_max_ = 0.17 e Å^−^^3^
0 restraints	Δρ_min_ = −0.12 e Å^−^^3^

### Special details

**Table:**

Geometry. All e.s.d.'s (except the e.s.d. in the dihedral angle between two l.s. planes) are estimated using the full covariance matrix. The cell e.s.d.'s are taken into account individually in the estimation of e.s.d.'s in distances, angles and torsion angles; correlations between e.s.d.'s in cell parameters are only used when they are defined by crystal symmetry. An approximate (isotropic) treatment of cell e.s.d.'s is used for estimating e.s.d.'s involving l.s. planes.
Refinement. Refinement of *F*^2^ against ALL reflections. The weighted *R*-factor *wR* and goodness of fit *S* are based on *F*^2^, conventional *R*-factors *R* are based on *F*, with *F* set to zero for negative *F*^2^. The threshold expression of *F*^2^ > σ(*F*^2^) is used only for calculating *R*-factors(gt) *etc*. and is not relevant to the choice of reflections for refinement. *R*-factors based on *F*^2^ are statistically about twice as large as those based on *F*, and *R*- factors based on ALL data will be even larger.

### Fractional atomic coordinates and isotropic or equivalent isotropic displacement parameters (Å^2^)

**Table:**

	*x*	*y*	*z*	*U*_iso_*/*U*_eq_	
C1	0.28649 (18)	0.60650 (15)	0.14800 (13)	0.0465 (5)	
C2	0.39947 (18)	0.55813 (15)	0.17139 (13)	0.0477 (5)	
C3	0.43181 (18)	0.54106 (16)	0.26117 (13)	0.0492 (5)	
C4	0.34696 (19)	0.57713 (16)	0.32313 (13)	0.0520 (5)	
H4A	0.3666	0.5672	0.3827	0.062*	
C5	0.23390 (18)	0.62743 (15)	0.30273 (13)	0.0489 (5)	
C6	0.20550 (18)	0.64095 (16)	0.21424 (13)	0.0497 (5)	
H6	0.1310	0.6736	0.1981	0.060*	
C7	0.2566 (2)	0.62093 (16)	0.05325 (14)	0.0524 (5)	
C8	0.5538 (2)	0.48601 (18)	0.28850 (14)	0.0587 (6)	
C9	0.5530 (3)	0.3694 (2)	0.23936 (18)	0.0877 (8)	
H9A	0.5513	0.3731	0.1759	0.131*	
H9B	0.4793	0.3274	0.2529	0.131*	
H9C	0.6281	0.3348	0.2585	0.131*	
C10	0.6714 (2)	0.5544 (3)	0.26778 (19)	0.0925 (9)	
H10A	0.6688	0.5606	0.2047	0.139*	
H10B	0.7467	0.5190	0.2854	0.139*	
H10C	0.6723	0.6264	0.3003	0.139*	
C11	0.5654 (2)	0.4759 (2)	0.38918 (15)	0.0798 (7)	
H11A	0.6425	0.4416	0.4041	0.120*	
H11B	0.4941	0.4321	0.4045	0.120*	
H11C	0.5668	0.5478	0.4221	0.120*	
C12	0.1488 (2)	0.66550 (18)	0.37777 (14)	0.0579 (6)	
C13	0.0264 (3)	0.7094 (3)	0.34080 (18)	0.0984 (10)	
H13A	0.0461	0.7705	0.3084	0.148*	
H13B	−0.0240	0.7331	0.3894	0.148*	
H13C	−0.0204	0.6524	0.3013	0.148*	
C14	0.1143 (3)	0.5700 (2)	0.42967 (18)	0.0903 (8)	
H14A	0.0661	0.5136	0.3904	0.135*	
H14B	0.0645	0.5953	0.4781	0.135*	
H14C	0.1905	0.5407	0.4533	0.135*	
C15	0.2221 (3)	0.7569 (2)	0.4411 (2)	0.1051 (10)	
H15A	0.2982	0.7290	0.4663	0.158*	
H15B	0.1701	0.7818	0.4884	0.158*	
H15C	0.2439	0.8171	0.4083	0.158*	
C16	0.1065 (2)	0.67878 (19)	−0.05259 (14)	0.0563 (5)	
C17	0.1612 (2)	0.7639 (2)	−0.08992 (16)	0.0721 (7)	
H17	0.2260	0.8094	−0.0584	0.086*	
C18	0.1182 (3)	0.7812 (2)	−0.17584 (17)	0.0811 (7)	
H18	0.1549	0.8385	−0.2027	0.097*	
C19	0.0230 (3)	0.7153 (2)	−0.22116 (17)	0.0859 (8)	
H19	−0.0048	0.7269	−0.2791	0.103*	
C20	−0.0319 (3)	0.6321 (2)	−0.18181 (18)	0.0956 (9)	
H20	−0.0986	0.5881	−0.2124	0.115*	
C21	0.0109 (2)	0.6127 (2)	−0.09681 (17)	0.0801 (7)	
H21	−0.0254	0.5551	−0.0702	0.096*	
C22	0.12793 (17)	0.97781 (16)	0.23838 (13)	0.0473 (5)	
C23	0.13758 (18)	1.04161 (16)	0.32194 (13)	0.0496 (5)	
C24	0.25789 (19)	1.07809 (16)	0.36367 (13)	0.0505 (5)	
C25	0.36198 (19)	1.04615 (16)	0.31783 (13)	0.0519 (5)	
H25	0.4421	1.0689	0.3447	0.062*	
C26	0.35629 (18)	0.98219 (15)	0.23419 (13)	0.0490 (5)	
C27	0.23656 (18)	0.94917 (16)	0.19592 (13)	0.0485 (5)	
H27	0.2282	0.9068	0.1404	0.058*	
C28	0.00075 (19)	0.93956 (16)	0.19638 (14)	0.0514 (5)	
C29	0.2727 (2)	1.14813 (18)	0.45578 (14)	0.0600 (6)	
C30	0.2086 (2)	1.25680 (19)	0.44900 (17)	0.0786 (7)	
H30A	0.2464	1.2951	0.4043	0.118*	
H30B	0.2196	1.3014	0.5059	0.118*	
H30C	0.1196	1.2419	0.4326	0.118*	
C31	0.2142 (2)	1.0855 (2)	0.52725 (15)	0.0811 (7)	
H31A	0.1257	1.0680	0.5105	0.122*	
H31B	0.2233	1.1305	0.5840	0.122*	
H31C	0.2570	1.0188	0.5320	0.122*	
C32	0.4136 (2)	1.1771 (2)	0.48629 (16)	0.0781 (7)	
H32A	0.4566	1.1107	0.4919	0.117*	
H32B	0.4196	1.2216	0.5432	0.117*	
H32C	0.4523	1.2169	0.4428	0.117*	
C33	0.47866 (18)	0.94936 (17)	0.19175 (14)	0.0564 (6)	
C34	0.5428 (2)	0.8673 (2)	0.24641 (18)	0.0914 (9)	
H34A	0.5688	0.9024	0.3054	0.137*	
H34B	0.6158	0.8415	0.2176	0.137*	
H34C	0.4841	0.8061	0.2507	0.137*	
C35	0.5688 (2)	1.0500 (2)	0.1888 (2)	0.0921 (9)	
H35A	0.5279	1.1012	0.1541	0.138*	
H35B	0.6445	1.0276	0.1618	0.138*	
H35C	0.5905	1.0845	0.2487	0.138*	
C36	0.4521 (2)	0.8948 (2)	0.09531 (16)	0.0810 (7)	
H36A	0.4005	0.8282	0.0951	0.122*	
H36B	0.5309	0.8781	0.0701	0.122*	
H36C	0.4082	0.9440	0.0603	0.122*	
C37	−0.10673 (18)	0.83525 (18)	0.06791 (14)	0.0545 (5)	
C38	−0.1617 (2)	0.74454 (19)	0.09664 (15)	0.0671 (6)	
H38	−0.1317	0.7198	0.1499	0.081*	
C39	−0.2638 (2)	0.6893 (2)	0.04492 (17)	0.0752 (7)	
H39	−0.3031	0.6271	0.0635	0.090*	
C40	−0.3064 (2)	0.7267 (2)	−0.03333 (17)	0.0750 (7)	
H40	−0.3749	0.6900	−0.0679	0.090*	
C41	−0.2489 (2)	0.8176 (2)	−0.06085 (17)	0.0782 (7)	
H41	−0.2779	0.8421	−0.1144	0.094*	
C42	−0.1484 (2)	0.87318 (19)	−0.01006 (16)	0.0678 (6)	
H42	−0.1093	0.9356	−0.0285	0.081*	
O1	0.48062 (13)	0.52679 (12)	0.10891 (9)	0.0666 (4)	
H1	0.4516	0.5399	0.0596	0.100*	
O2	0.32547 (15)	0.59790 (14)	−0.00665 (10)	0.0731 (5)	
O3	0.14275 (14)	0.66219 (13)	0.03719 (9)	0.0661 (4)	
O4	0.03285 (13)	1.06905 (12)	0.36496 (10)	0.0678 (4)	
H4	−0.0306	1.0390	0.3364	0.102*	
O5	−0.09842 (14)	0.95344 (13)	0.23141 (10)	0.0669 (4)	
O6	0.00567 (13)	0.88675 (13)	0.11387 (10)	0.0666 (4)	

### Atomic displacement parameters (Å^2^)

**Table:**

	*U*^11^	*U*^22^	*U*^33^	*U*^12^	*U*^13^	*U*^23^
C1	0.0491 (12)	0.0432 (11)	0.0472 (12)	0.0004 (9)	0.0068 (9)	0.0040 (9)
C2	0.0475 (12)	0.0450 (12)	0.0509 (12)	0.0014 (9)	0.0123 (10)	0.0015 (9)
C3	0.0493 (12)	0.0464 (12)	0.0512 (12)	0.0012 (9)	0.0036 (10)	0.0034 (9)
C4	0.0572 (13)	0.0521 (13)	0.0464 (12)	0.0001 (10)	0.0019 (10)	0.0069 (10)
C5	0.0543 (13)	0.0459 (12)	0.0473 (12)	0.0017 (10)	0.0080 (9)	0.0068 (9)
C6	0.0458 (11)	0.0493 (12)	0.0552 (13)	0.0050 (9)	0.0053 (9)	0.0090 (10)
C7	0.0574 (13)	0.0492 (13)	0.0507 (13)	0.0018 (10)	0.0068 (11)	0.0049 (10)
C8	0.0543 (13)	0.0634 (14)	0.0575 (13)	0.0105 (11)	−0.0014 (10)	0.0038 (11)
C9	0.0928 (19)	0.0795 (18)	0.0883 (19)	0.0381 (15)	−0.0122 (15)	−0.0038 (15)
C10	0.0503 (15)	0.123 (2)	0.106 (2)	−0.0012 (14)	−0.0013 (13)	0.0260 (18)
C11	0.0799 (17)	0.0903 (19)	0.0696 (17)	0.0216 (14)	−0.0084 (13)	0.0117 (14)
C12	0.0620 (14)	0.0644 (14)	0.0506 (12)	0.0107 (11)	0.0144 (10)	0.0115 (11)
C13	0.090 (2)	0.138 (3)	0.0796 (18)	0.0518 (19)	0.0366 (15)	0.0316 (17)
C14	0.100 (2)	0.100 (2)	0.0811 (18)	0.0157 (16)	0.0397 (15)	0.0321 (16)
C15	0.111 (2)	0.101 (2)	0.096 (2)	−0.0011 (18)	0.0316 (18)	−0.0324 (17)
C16	0.0592 (13)	0.0633 (14)	0.0469 (12)	0.0070 (11)	0.0015 (10)	0.0093 (11)
C17	0.0699 (16)	0.0745 (17)	0.0712 (16)	−0.0074 (13)	−0.0078 (12)	0.0183 (13)
C18	0.0847 (18)	0.0844 (18)	0.0784 (18)	−0.0052 (15)	−0.0023 (15)	0.0365 (15)
C19	0.102 (2)	0.092 (2)	0.0635 (16)	−0.0005 (17)	−0.0178 (15)	0.0251 (15)
C20	0.117 (2)	0.088 (2)	0.0746 (18)	−0.0269 (17)	−0.0302 (16)	0.0170 (16)
C21	0.0974 (19)	0.0727 (17)	0.0687 (16)	−0.0194 (14)	−0.0103 (14)	0.0219 (13)
C22	0.0419 (11)	0.0487 (12)	0.0511 (12)	−0.0020 (9)	0.0008 (9)	0.0079 (9)
C23	0.0445 (12)	0.0524 (12)	0.0528 (12)	0.0011 (9)	0.0067 (10)	0.0082 (10)
C24	0.0491 (12)	0.0516 (12)	0.0499 (12)	−0.0010 (10)	0.0004 (10)	0.0051 (10)
C25	0.0458 (12)	0.0541 (13)	0.0534 (12)	−0.0059 (9)	−0.0044 (10)	0.0040 (10)
C26	0.0465 (12)	0.0471 (12)	0.0531 (12)	−0.0034 (9)	0.0026 (9)	0.0083 (10)
C27	0.0481 (12)	0.0497 (12)	0.0464 (11)	−0.0018 (9)	0.0022 (9)	0.0035 (9)
C28	0.0473 (13)	0.0517 (13)	0.0554 (13)	−0.0019 (10)	0.0039 (10)	0.0080 (10)
C29	0.0639 (14)	0.0613 (14)	0.0524 (13)	0.0021 (11)	−0.0011 (10)	−0.0007 (11)
C30	0.0900 (18)	0.0670 (16)	0.0741 (16)	0.0089 (13)	−0.0019 (14)	−0.0093 (13)
C31	0.0917 (19)	0.097 (2)	0.0540 (14)	−0.0001 (15)	0.0044 (13)	0.0074 (13)
C32	0.0756 (17)	0.0820 (17)	0.0690 (16)	−0.0014 (13)	−0.0111 (13)	−0.0137 (13)
C33	0.0455 (12)	0.0562 (13)	0.0666 (14)	−0.0007 (10)	0.0067 (10)	0.0030 (11)
C34	0.0792 (18)	0.103 (2)	0.097 (2)	0.0371 (16)	0.0107 (15)	0.0182 (17)
C35	0.0641 (16)	0.0845 (19)	0.125 (2)	−0.0144 (14)	0.0310 (15)	−0.0090 (17)
C36	0.0643 (16)	0.103 (2)	0.0730 (17)	0.0057 (14)	0.0162 (12)	−0.0098 (14)
C37	0.0394 (11)	0.0659 (14)	0.0553 (13)	−0.0075 (10)	0.0004 (10)	−0.0003 (11)
C38	0.0653 (15)	0.0770 (17)	0.0575 (14)	−0.0098 (13)	−0.0042 (11)	0.0129 (12)
C39	0.0727 (16)	0.0735 (17)	0.0770 (17)	−0.0200 (13)	0.0010 (14)	0.0104 (13)
C40	0.0592 (15)	0.0844 (19)	0.0752 (17)	−0.0148 (13)	−0.0154 (12)	0.0018 (14)
C41	0.0771 (17)	0.0809 (18)	0.0742 (16)	−0.0055 (14)	−0.0209 (13)	0.0182 (14)
C42	0.0648 (15)	0.0634 (15)	0.0736 (16)	−0.0117 (12)	−0.0096 (12)	0.0165 (12)
O1	0.0627 (9)	0.0829 (11)	0.0561 (9)	0.0173 (8)	0.0136 (7)	0.0047 (8)
O2	0.0808 (11)	0.0938 (12)	0.0486 (9)	0.0266 (9)	0.0174 (8)	0.0093 (8)
O3	0.0598 (9)	0.0918 (11)	0.0493 (9)	0.0173 (8)	0.0047 (7)	0.0145 (8)
O4	0.0505 (9)	0.0833 (11)	0.0667 (10)	0.0006 (8)	0.0095 (7)	−0.0061 (8)
O5	0.0442 (9)	0.0794 (11)	0.0739 (10)	−0.0023 (7)	0.0051 (7)	−0.0037 (8)
O6	0.0474 (9)	0.0892 (11)	0.0579 (9)	−0.0118 (8)	0.0004 (7)	−0.0066 (8)

### Geometric parameters (Å, °)

**Table:**

C1—C6	1.400 (3)	C22—C27	1.391 (3)
C1—C2	1.400 (3)	C22—C23	1.400 (3)
C1—C7	1.472 (3)	C22—C28	1.479 (3)
C2—O1	1.351 (2)	C23—O4	1.356 (2)
C2—C3	1.413 (3)	C23—C24	1.410 (3)
O1—H1	0.8200	O4—H4	0.8200
C3—C4	1.388 (3)	C24—C25	1.386 (3)
C3—C8	1.537 (3)	C24—C29	1.542 (3)
C8—C9	1.534 (3)	C28—O5	1.215 (2)
C8—C11	1.532 (3)	C28—O6	1.340 (2)
C8—C10	1.535 (3)	C29—C30	1.533 (3)
C9—H9A	0.9600	C29—C31	1.535 (3)
C9—H9B	0.9600	C29—C32	1.542 (3)
C9—H9C	0.9600	C30—H30A	0.9600
C10—H10A	0.9600	C30—H30B	0.9600
C10—H10B	0.9600	C30—H30C	0.9600
C10—H10C	0.9600	C31—H31A	0.9600
C11—H11A	0.9600	C31—H31B	0.9600
C11—H11B	0.9600	C31—H31C	0.9600
C11—H11C	0.9600	C25—C26	1.403 (3)
C4—C5	1.402 (3)	C25—H25	0.9300
C4—H4A	0.9300	C26—C27	1.378 (3)
C5—C6	1.376 (3)	C26—C33	1.531 (3)
C5—C12	1.536 (3)	C27—H27	0.9300
C12—C13	1.520 (3)	C32—H32A	0.9600
C12—C14	1.522 (3)	C32—H32B	0.9600
C12—C15	1.531 (3)	C32—H32C	0.9600
C13—H13A	0.9600	C33—C34	1.525 (3)
C13—H13B	0.9600	C33—C35	1.529 (3)
C13—H13C	0.9600	C33—C36	1.531 (3)
C14—H14A	0.9600	C34—H34A	0.9600
C14—H14B	0.9600	C34—H34B	0.9600
C14—H14C	0.9600	C34—H34C	0.9600
C15—H15A	0.9600	C35—H35A	0.9600
C15—H15B	0.9600	C35—H35B	0.9600
C15—H15C	0.9600	C35—H35C	0.9600
C6—H6	0.9300	O6—C37	1.418 (2)
C7—O2	1.213 (2)	C36—H36A	0.9600
C7—O3	1.345 (2)	C36—H36B	0.9600
O3—C16	1.419 (2)	C36—H36C	0.9600
C16—C21	1.354 (3)	C37—C38	1.357 (3)
C16—C17	1.362 (3)	C37—C42	1.364 (3)
C17—C18	1.384 (3)	C38—C39	1.389 (3)
C17—H17	0.9300	C38—H38	0.9300
C18—C19	1.357 (3)	C39—C40	1.368 (3)
C18—H18	0.9300	C39—H39	0.9300
C19—C20	1.361 (3)	C40—C41	1.361 (3)
C19—H19	0.9300	C40—H40	0.9300
C20—C21	1.378 (3)	C41—C42	1.373 (3)
C20—H20	0.9300	C41—H41	0.9300
C21—H21	0.9300	C42—H42	0.9300
			
C6—C1—C2	119.99 (18)	C27—C22—C23	120.52 (18)
C6—C1—C7	121.49 (18)	C27—C22—C28	120.31 (18)
C2—C1—C7	118.51 (17)	C23—C22—C28	119.17 (18)
O1—C2—C1	121.01 (18)	O4—C23—C22	121.38 (18)
O1—C2—C3	118.21 (17)	O4—C23—C24	118.46 (18)
C1—C2—C3	120.78 (17)	C22—C23—C24	120.16 (18)
C2—O1—H1	109.5	C23—O4—H4	109.5
C4—C3—C2	116.01 (18)	C25—C24—C23	116.26 (18)
C4—C3—C8	122.06 (18)	C25—C24—C29	121.95 (18)
C2—C3—C8	121.93 (17)	C23—C24—C29	121.78 (18)
C9—C8—C11	107.30 (19)	O5—C28—O6	122.67 (18)
C9—C8—C10	110.2 (2)	O5—C28—C22	124.8 (2)
C11—C8—C10	107.08 (19)	O6—C28—C22	112.52 (18)
C9—C8—C3	110.12 (18)	C30—C29—C31	110.5 (2)
C11—C8—C3	111.52 (17)	C30—C29—C24	109.31 (17)
C10—C8—C3	110.54 (19)	C31—C29—C24	110.49 (18)
C8—C9—H9A	109.5	C30—C29—C32	107.23 (19)
C8—C9—H9B	109.5	C31—C29—C32	107.82 (18)
H9A—C9—H9B	109.5	C24—C29—C32	111.45 (18)
C8—C9—H9C	109.5	C29—C30—H30A	109.5
H9A—C9—H9C	109.5	C29—C30—H30B	109.5
H9B—C9—H9C	109.5	H30A—C30—H30B	109.5
C8—C10—H10A	109.5	C29—C30—H30C	109.5
C8—C10—H10B	109.5	H30A—C30—H30C	109.5
H10A—C10—H10B	109.5	H30B—C30—H30C	109.5
C8—C10—H10C	109.5	C29—C31—H31A	109.5
H10A—C10—H10C	109.5	C29—C31—H31B	109.5
H10B—C10—H10C	109.5	H31A—C31—H31B	109.5
C8—C11—H11A	109.5	C29—C31—H31C	109.5
C8—C11—H11B	109.5	H31A—C31—H31C	109.5
H11A—C11—H11B	109.5	H31B—C31—H31C	109.5
C8—C11—H11C	109.5	C24—C25—C26	125.29 (18)
H11A—C11—H11C	109.5	C24—C25—H25	117.4
H11B—C11—H11C	109.5	C26—C25—H25	117.4
C3—C4—C5	124.99 (18)	C27—C26—C25	116.23 (18)
C3—C4—H4A	117.5	C27—C26—C33	123.47 (18)
C5—C4—H4A	117.5	C25—C26—C33	120.26 (18)
C6—C5—C4	116.93 (18)	C26—C27—C22	121.53 (19)
C6—C5—C12	123.04 (18)	C26—C27—H27	119.2
C4—C5—C12	120.03 (17)	C22—C27—H27	119.2
C13—C12—C14	108.2 (2)	C29—C32—H32A	109.5
C13—C12—C15	109.0 (2)	C29—C32—H32B	109.5
C14—C12—C15	109.5 (2)	H32A—C32—H32B	109.5
C13—C12—C5	111.69 (18)	C29—C32—H32C	109.5
C14—C12—C5	110.08 (18)	H32A—C32—H32C	109.5
C15—C12—C5	108.38 (18)	H32B—C32—H32C	109.5
C12—C13—H13A	109.5	C34—C33—C35	110.0 (2)
C12—C13—H13B	109.5	C34—C33—C36	108.1 (2)
H13A—C13—H13B	109.5	C35—C33—C36	107.2 (2)
C12—C13—H13C	109.5	C34—C33—C26	108.84 (18)
H13A—C13—H13C	109.5	C35—C33—C26	110.91 (17)
H13B—C13—H13C	109.5	C36—C33—C26	111.75 (17)
C12—C14—H14A	109.5	C33—C34—H34A	109.5
C12—C14—H14B	109.5	C33—C34—H34B	109.5
H14A—C14—H14B	109.5	H34A—C34—H34B	109.5
C12—C14—H14C	109.5	C33—C34—H34C	109.5
H14A—C14—H14C	109.5	H34A—C34—H34C	109.5
H14B—C14—H14C	109.5	H34B—C34—H34C	109.5
C12—C15—H15A	109.5	C33—C35—H35A	109.5
C12—C15—H15B	109.5	C33—C35—H35B	109.5
H15A—C15—H15B	109.5	H35A—C35—H35B	109.5
C12—C15—H15C	109.5	C33—C35—H35C	109.5
H15A—C15—H15C	109.5	H35A—C35—H35C	109.5
H15B—C15—H15C	109.5	H35B—C35—H35C	109.5
C5—C6—C1	121.29 (18)	C28—O6—C37	119.58 (16)
C5—C6—H6	119.4	C33—C36—H36A	109.5
C1—C6—H6	119.4	C33—C36—H36B	109.5
O2—C7—O3	121.45 (19)	H36A—C36—H36B	109.5
O2—C7—C1	124.78 (19)	C33—C36—H36C	109.5
O3—C7—C1	113.76 (18)	H36A—C36—H36C	109.5
C7—O3—C16	117.41 (15)	H36B—C36—H36C	109.5
C21—C16—C17	121.7 (2)	C38—C37—C42	122.0 (2)
C21—C16—O3	118.0 (2)	C38—C37—O6	120.11 (19)
C17—C16—O3	120.1 (2)	C42—C37—O6	117.56 (19)
C16—C17—C18	118.4 (2)	C37—C38—C39	118.7 (2)
C16—C17—H17	120.8	C37—C38—H38	120.7
C18—C17—H17	120.8	C39—C38—H38	120.7
C19—C18—C17	120.5 (2)	C40—C39—C38	119.8 (2)
C19—C18—H18	119.7	C40—C39—H39	120.1
C17—C18—H18	119.7	C38—C39—H39	120.1
C18—C19—C20	120.0 (2)	C41—C40—C39	120.3 (2)
C18—C19—H19	120.0	C41—C40—H40	119.9
C20—C19—H19	120.0	C39—C40—H40	119.9
C19—C20—C21	120.3 (2)	C40—C41—C42	120.4 (2)
C19—C20—H20	119.9	C40—C41—H41	119.8
C21—C20—H20	119.9	C42—C41—H41	119.8
C16—C21—C20	119.1 (2)	C37—C42—C41	118.8 (2)
C16—C21—H21	120.5	C37—C42—H42	120.6
C20—C21—H21	120.5	C41—C42—H42	120.6
			
C6—C1—C2—O1	−178.32 (17)	C27—C22—C23—O4	−179.05 (17)
C7—C1—C2—O1	1.0 (3)	C28—C22—C23—O4	−0.1 (3)
C6—C1—C2—C3	1.5 (3)	C27—C22—C23—C24	0.4 (3)
C7—C1—C2—C3	−179.15 (17)	C28—C22—C23—C24	179.38 (17)
O1—C2—C3—C4	178.44 (17)	O4—C23—C24—C25	178.80 (17)
C1—C2—C3—C4	−1.4 (3)	C22—C23—C24—C25	−0.7 (3)
O1—C2—C3—C8	−1.4 (3)	O4—C23—C24—C29	−0.3 (3)
C1—C2—C3—C8	178.72 (18)	C22—C23—C24—C29	−179.78 (18)
C4—C3—C8—C9	119.9 (2)	C27—C22—C28—O5	174.2 (2)
C2—C3—C8—C9	−60.2 (3)	C23—C22—C28—O5	−4.8 (3)
C4—C3—C8—C11	0.9 (3)	C27—C22—C28—O6	−5.8 (3)
C2—C3—C8—C11	−179.18 (19)	C23—C22—C28—O6	175.22 (17)
C4—C3—C8—C10	−118.1 (2)	C25—C24—C29—C30	119.3 (2)
C2—C3—C8—C10	61.8 (3)	C23—C24—C29—C30	−61.6 (3)
C2—C3—C4—C5	0.4 (3)	C25—C24—C29—C31	−118.9 (2)
C8—C3—C4—C5	−179.69 (18)	C23—C24—C29—C31	60.2 (3)
C3—C4—C5—C6	0.5 (3)	C25—C24—C29—C32	0.9 (3)
C3—C4—C5—C12	−179.01 (18)	C23—C24—C29—C32	−179.98 (19)
C6—C5—C12—C13	5.1 (3)	C23—C24—C25—C26	0.6 (3)
C4—C5—C12—C13	−175.5 (2)	C29—C24—C25—C26	179.70 (18)
C6—C5—C12—C14	125.3 (2)	C24—C25—C26—C27	−0.2 (3)
C4—C5—C12—C14	−55.3 (3)	C24—C25—C26—C33	−178.11 (18)
C6—C5—C12—C15	−115.0 (2)	C25—C26—C27—C22	−0.1 (3)
C4—C5—C12—C15	64.4 (3)	C33—C26—C27—C22	177.72 (18)
C4—C5—C6—C1	−0.4 (3)	C23—C22—C27—C26	0.0 (3)
C12—C5—C6—C1	179.08 (18)	C28—C22—C27—C26	−178.95 (18)
C2—C1—C6—C5	−0.6 (3)	C27—C26—C33—C34	−108.6 (2)
C7—C1—C6—C5	−179.91 (18)	C25—C26—C33—C34	69.1 (2)
C6—C1—C7—O2	176.21 (19)	C27—C26—C33—C35	130.3 (2)
C2—C1—C7—O2	−3.1 (3)	C25—C26—C33—C35	−52.0 (3)
C6—C1—C7—O3	−4.3 (3)	C27—C26—C33—C36	10.7 (3)
C2—C1—C7—O3	176.35 (17)	C25—C26—C33—C36	−171.57 (19)
O2—C7—O3—C16	−0.6 (3)	O5—C28—O6—C37	−6.3 (3)
C1—C7—O3—C16	179.95 (17)	C22—C28—O6—C37	173.70 (17)
C7—O3—C16—C21	110.4 (2)	C28—O6—C37—C38	−69.2 (3)
C7—O3—C16—C17	−74.1 (3)	C28—O6—C37—C42	117.4 (2)
C21—C16—C17—C18	−1.0 (4)	C42—C37—C38—C39	−0.3 (3)
O3—C16—C17—C18	−176.3 (2)	O6—C37—C38—C39	−173.3 (2)
C16—C17—C18—C19	0.6 (4)	C37—C38—C39—C40	0.2 (4)
C17—C18—C19—C20	0.7 (4)	C38—C39—C40—C41	0.2 (4)
C18—C19—C20—C21	−1.6 (5)	C39—C40—C41—C42	−0.6 (4)
C17—C16—C21—C20	0.1 (4)	C38—C37—C42—C41	−0.1 (4)
O3—C16—C21—C20	175.6 (2)	O6—C37—C42—C41	173.2 (2)
C19—C20—C21—C16	1.2 (4)	C40—C41—C42—C37	0.5 (4)

### Hydrogen-bond geometry (Å, °)

**Table:**

*D*—H···*A*	*D*—H	H···*A*	*D*···*A*	*D*—H···*A*
O1—H1···O2	0.82	1.83	2.563 (2)	148
O4—H4···O5	0.82	1.88	2.604 (2)	147
